# Supplementary material for: Getting More Out of Biomedical Documents with GATE's Full Lifecycle Open Source Text Analytics
Source: PLoS Comput Biol. 2013 Feb 7;9(2):e1002854. doi: 10.1371/journal.pcbi.1002854 (PMC3567135; doi:10.1371/journal.pcbi.1002854)
Supplement: Dataset S1 — GATE software. Dataset S1 bundled with this paper contains a distribution of GATE (or see http://gate.ac.uk/download/). (TGZ) [file pcbi.1002854.s001.tgz › plos-gate/plugins/Parser_RASP/doc/modules.html]

RASP2 plugin for GATE

  

# RASP2 plugin for GATE

Previous: Overview and Installation

## RASP2 modules

The RASP2 plugin for GATE provides 4 Processing Resources, each one of
them requires the annotation types from the previous ones.  
http://www.informatics.sussex.ac.uk/research/groups/nlp/rasp/offline-demo.html contains a description of the original modules in RASP.

### Tokenizer

Creates annotations of type Token, using the information about Sentences. A Token is a simple annotation which contains only a feature 'string'
(unlike the default component in GATE which has more information).
Another difference with the GATE equivalent is that the Tokenizer
requires annotations of type Sentence and hence can't be the first
element of a pipeline. Check out the resources pages on the
DigitalPebble website for our Toolbox which contains a Sentence
Splitter. The original GATE tokenizer can be used instead of the RASP
one.  
  
Runtime Parameters  
  

|  |  |
| --- | --- |
| inputASName | AnnotationSet where the Sentences are taken from |
| outputASName | AnnotationSet where the Tokens are generated |
| debug | Keeps the file returned by the Tokenizer in the /tmp directory |
| charset | Specifies the charset to use for the communication with the Tokenizer |

  

### POS Tagger

The Part of Speech Tagger generates annotations of type WordForms. This separation between Tokens and WForms is based on the MAF ISO proposal.   
The tagset is close to CLAWS C7 (see e.g. Appendix C of Jurafsky,
D. and Martin, J. Speech and Language Processing, Prentice-Hall, 2000
for more details), although it is in fact a cut down version of the
CLAWS C2 tagset.  
  
A WordForm gets a POS attribute, which is a simple String and a probability.  
  
Init Parameter  
  

|  |  |
| --- | --- |
| raspHome | Directory where RASP is installed |

  
Runtime Parameters  
  

|  |  |
| --- | --- |
| inputASName | AnnotationSet where Sentences and Tokens are taken from |
| outputASName | AnnotationSet where the WordForms are generated |
| debug | Keeps the file returned by the POSTagger in the /tmp directory |
| charset | Specifies the charset to use for the communication with the POSTagger |
| generateMultipleTags | Generates one or more WordForms per Token |

  

### POS Converter

The Part of Speech Converter achieves
a similar functionality as above but instead of getting the POS tags
from the original RASP component, it converts the tags generated by the
default GATE POS tagger (PennTreebank tagset) into the tagset used by
RASP. This has an advantage of speeding up the processing at the cost
of possible inaccuracies in the conversion.  
  
Runtime Parameters  
  

|  |  |
| --- | --- |
| inputASName | AnnotationSet where WordForms are taken from |
| outputASName | AnnotationSet where the WordForms are generated |
| grammarURL | URL to the JAPE grammar file |
| encoding | The encoding used for reading the grammar |

  

### Morphological Analyser

Next the tagger output is lemmatized, based on the tags
assigned to word tokens. See Briscoe and Carroll (2002) for further details
and a reference to a detailed paper describing this module. The Morphological Analyzer adds an attribute lemma to the WordForms found in the input AnnotationSet.  
  
Init Parameter  
  

|  |  |
| --- | --- |
| raspHome | Directory where RASP is installed |

  
Runtime Parameters  
  

|  |  |
| --- | --- |
| inputASName | AnnotationSet where WordForms are taken from |
| debug | Keeps the file returned by the Morpher in the /tmp directory |
| charset | Specifies the charset to use for the communication with the Morpher |

  


### Parser

The probabilistic parser analyses the PoS tag sequence or
chart of initial more probable tags and generates a parse forest
representation containing all possible subanalyses with associated
probabilities. From this representation it is able to construct
the n-best (weighted) grammatical relations.  
  
The parser generates annotations of the type Dependency. Dependencies have a type and subtype and link to two WordForms as head and dependency. This implementation does not generate annotations for Clauses.  
  
Note that the Parser requires a recent machine with minimum 1.5G of RAM.  
  
Init Parameter  
  

|  |  |
| --- | --- |
| raspHome | Directory where RASP is installed |

  
Runtime Parameters  
  

|  |  |
| --- | --- |
| inputASName | AnnotationSet where WordForms and Sentences are taken from |
| outputASName | AnnotationSet where the Dependencies are generated |
| debug | Keeps the file returned by the Parser in the /tmp directory |
| charset | Specifies the charset to use for the communication with the Parser |
| subcategorisation | Turns subcategorisation on or off |
| phrasalVerbs | Turns the use of phrasal verbs on or off |
| outputFormat | Format returned by the Parser. Authorized values are "-og","-ogio", "-ogw". See RASP documentation for more information. |

  
  
  
 
